# Early-stage squamous cell carcinoma of the oropharynx: Radiotherapy vs. Trans-Oral Robotic Surgery (ORATOR) – study protocol for a randomized phase II trial

**DOI:** 10.1186/1471-2407-13-133

**Published:** 2013-03-20

**Authors:** Anthony C Nichols, John Yoo, J Alex Hammond, Kevin Fung, Eric Winquist, Nancy Read, Varagur Venkatesan, S Danielle MacNeil, D Scott Ernst, Sara Kuruvilla, Jeff Chen, Martin Corsten, Michael Odell, Libni Eapen, Julie Theurer, Philip C Doyle, Bret Wehrli, Keith Kwan, David A Palma

**Affiliations:** 1 Department of Otolaryngology-Head and Neck Surgery, London Health Sciences Centre and Western University, London, Ontario, Canada; 2 Division of Radiation Oncology, Department of Oncology, London Health Sciences Centre and Western University, London, Ontario, Canada; 3 Division of Medical Oncology, Department of Oncology, London Health Sciences Centre and Western University, London, Ontario, Canada; 4 Department of Medical Biophysics, Western University, London, Ontario, Canada; 5 Department of Otolaryngology-Head and Neck Surgery, Ottawa Hospital, Ottawa, Ontario, Canada; 6 Division of Radiation Oncology, Ottawa Hospital Regional Cancer Centre, Ottawa, Ontario, Canada; 7 School of Communications Sciences and Disorders, Western University, London, Ontario, Canada; 8 Department of Pathology, London Health Sciences Centre, Western University, London, Ontario, Canada

**Keywords:** Head and neck cancer, Oropharynx, Human papillomavirus, Radiotherapy, Transoral robotic surgery, Quality of life, Survival, Randomized controlled trial

## Abstract

**Background:**

The incidence of oropharyngeal squamous cell carcinoma (OPSCC) has markedly increased over the last three decades due to newly found associations with human papillomavirus (HPV) infection. Primary radiotherapy (RT) is the treatment of choice for OPSCC at most centers, and over the last decade, the addition of concurrent chemotherapy has led to a significant improvement in survival, but at the cost of increased acute and late toxicity. Transoral robotic surgery (TORS) has emerged as a promising alternative treatment, with preliminary case series demonstrating encouraging oncologic, functional, and quality of life (QOL) outcomes. However, comparisons of TORS and RT in a non-randomized fashion are susceptible to bias. The goal of this randomized phase II study is to compare QOL, functional outcomes, toxicity profiles, and survival following primary RT (± chemotherapy) vs. TORS (± adjuvant [chemo] RT) in patients with OPSCC.

**Methods/Design:**

The target patient population comprises OPSCC patients who would be unlikely to require chemotherapy post-resection: Tumor stage T1-T2 with likely negative margins at surgery; Nodal stage N0-2, ≤3 cm in size, with no evidence of extranodal extension on imaging. Participants will be randomized in a 1:1 ratio between Arm 1 (RT ± chemotherapy) and Arm 2 (TORS ± adjuvant [chemo] RT). In Arm 1, patients with N0 disease will receive RT alone, whereas N1-2 patients will receive concurrent chemoradiation. In Arm 2, patients will undergo TORS along with selective neck dissections, which may be staged. Pathologic high-risk features will be used to determine the requirement for adjuvant radiotherapy +/- chemotherapy. The primary endpoint is QOL score using the M.D. Anderson Dysphagia Inventory (MDADI), with secondary endpoints including survival, toxicity, other QOL outcomes, and swallowing function. A sample of 68 patients is required.

**Discussion:**

This study, if successful, will provide a much-needed randomized comparison of the conventional strategy of primary RT vs. the novel strategy of primary TORS. The trial is designed to provide a definitive QOL comparison between the two arms, and to inform the design of an eventual phase III trial for survival outcomes.

**Trial registration:**

NCT01590355

## Background

The incidence of oropharyngeal squamous cell carcinoma (OPSCC) is rapidly increasing, associated with rising rates of oral infection with the human papillomavirus (HPV) [1]. Patients with HPV-related cancers tend to present at a younger age and experience markedly improved survival, compared to patients with smoking- and alcohol-related head and neck cancers [1]. In such young patients with good prognosis, survivorship is of paramount importance, as they are likely to survive their cancer and may have to cope with the side effects of therapy and secondary disability for decades. Optimizing post-treatment quality of life (QOL) for patients with OPSCC has become one of the most important issues in head and neck oncology.

Historically, oropharyngeal cancer was frequently managed with open surgery, with or without postoperative radiotherapy (RT). Although this approach was reasonably effective at obtaining tumor control, the speech, swallowing and cosmetic outcomes were poor. Surgical access of the oropharynx traditionally involves large facial and neck incisions that interrupted critical neurovascular and muscular structures [2]. This resulted in high complication rates, leading many centers to move towards organ preservation approaches utilizing radiation as primary treatment with surgery reserved for salvage [3]. Although a randomized trial was never carried out comparing surgery and RT, a meta-analysis of observational studies demonstrated that primary surgery and primary RT approaches were equivalent in terms of survival, but major complications of therapy were markedly worse in the primary surgery patients [2].

For patients with high-risk cancers of the oropharynx, concurrent cisplatin-based chemotherapy is delivered with RT and achieves a significant improvement in survival, compared to radiotherapy alone [4]. However, it is increasingly recognized that these improvements in survival come at the cost of increased acute and late toxicities: these include dysphagia, mucositis, xerostomia, fibrosis, osteoradionecrosis, neutropenia, neurotoxicity, nephrotoxicity and ototoxicity [5]. The addition of chemotherapy to RT increases the risk of long-term gastrostomy tube dependence from 1% to 13% [5]. These treatment-related toxicities can significantly affect QOL.

These toxicity risks, and their consequent reduction in QOL, have led to increasing interest in minimally invasive surgical approaches for OPSCC, especially transoral robotic surgery (TORS) using a surgical robot. The surgical robot is comprised of a binocular endoscope and two low profile articulating arms that can be placed in the oropharynx, while the surgeon sits at a separate console to control the instruments. This allows the operating surgeon to overcome the visualization and access challenges that can otherwise make transoral surgery in this area challenging, if not impossible. The ability to avoid incisions in the face and neck, which were required prior to the implementation of the robot, preserves neuromuscular structures that are critical for speech and swallowing. Preliminary case series of TORS have reported encouraging oncologic, functional, and QOL outcomes compared with primary RT [6,7].

Outcomes after TORS have been summarized in a recent systematic review [6]. All reports involve prospective or retrospective single-arm case series with varying use of adjuvant therapy without adequate controls. This is in stark contrast to the large number of randomized controlled trials of chemoradiation (CRT) for OPSCC. Favourable gastrostomy tube rates (0–9.5% at one year and 0% at two years post treatment) have been reported following TORS [6]. In contrast, feeding tube rates in CRT series range from 9 to 39% at one year [8,9]. Patients undergoing TORS experience excellent 2-year disease free survival (DFS) and overall survival (OS) rates of 79% to 89% and 82% to 90%, respectively [6]. CRT trials report 3-year DFS and OS rates of 42% to 76.5% and 51% to 85%, respectively [8,9]. Two studies have explored the impact of TORS on QOL. Patients receiving TORS alone report better health-related QOL compared to individuals receiving TORS plus adjuvant radiation or chemoradiation [7]. Swallowing-related QOL is reported to decrease immediately following TORS intervention, but has been demonstrated to improve by one year post-treatment, with possible further improvement thereafter [10].

Although these data would appear to favor a surgical approach, a careful review of the literature reveals that this is a tremendously biased comparison. The TORS studies include a much smaller fraction of T3/T4 tumors (0–30%) and N3 neck disease (0–4%) compared with CRT series (31–86% T3/T4 and 2.5–12% N3) [6,8,9]. There are numerous additional confounders that make these comparisons invalid including: 1) patients receiving surgery have been carefully selected (i.e. ‘confounding by indication’, which cannot be adequately controlled for in observational studies [11]), 2) each study contains a different fraction of HPV-positive tumors, and 3) the majority of TORS patients receive adjuvant therapy including radiation (24%) or chemoradiation (54%) [12], making the true benefits of TORS unclear. Not surprisingly, management of OPSCC is now one of the most contentious and important issues in head and neck oncology practice in the wake of the growing HPV OPSCC epidemic.

Given the rapid treatment paradigm shift in the absence of level I evidence, the high cost of acquiring a surgical robot, and increasing numbers of affected patients due to the HPV epidemic, a randomized trial is critical to guide the optimal management of OPSCC. The goal of this randomized phase II study is to formally compare QOL in patients with oropharyngeal cancer (T1-2, N0-2) after TORS vs. primary RT, and to inform the design of a large, definitive, comparative phase III trial assessing survival.

## Methods/Design

This study has been approved by the Ontario Cancer Research Ethics Board (#12-006), in compliance with the Helsinki Declaration.

### Objectives

**Figure 1:**
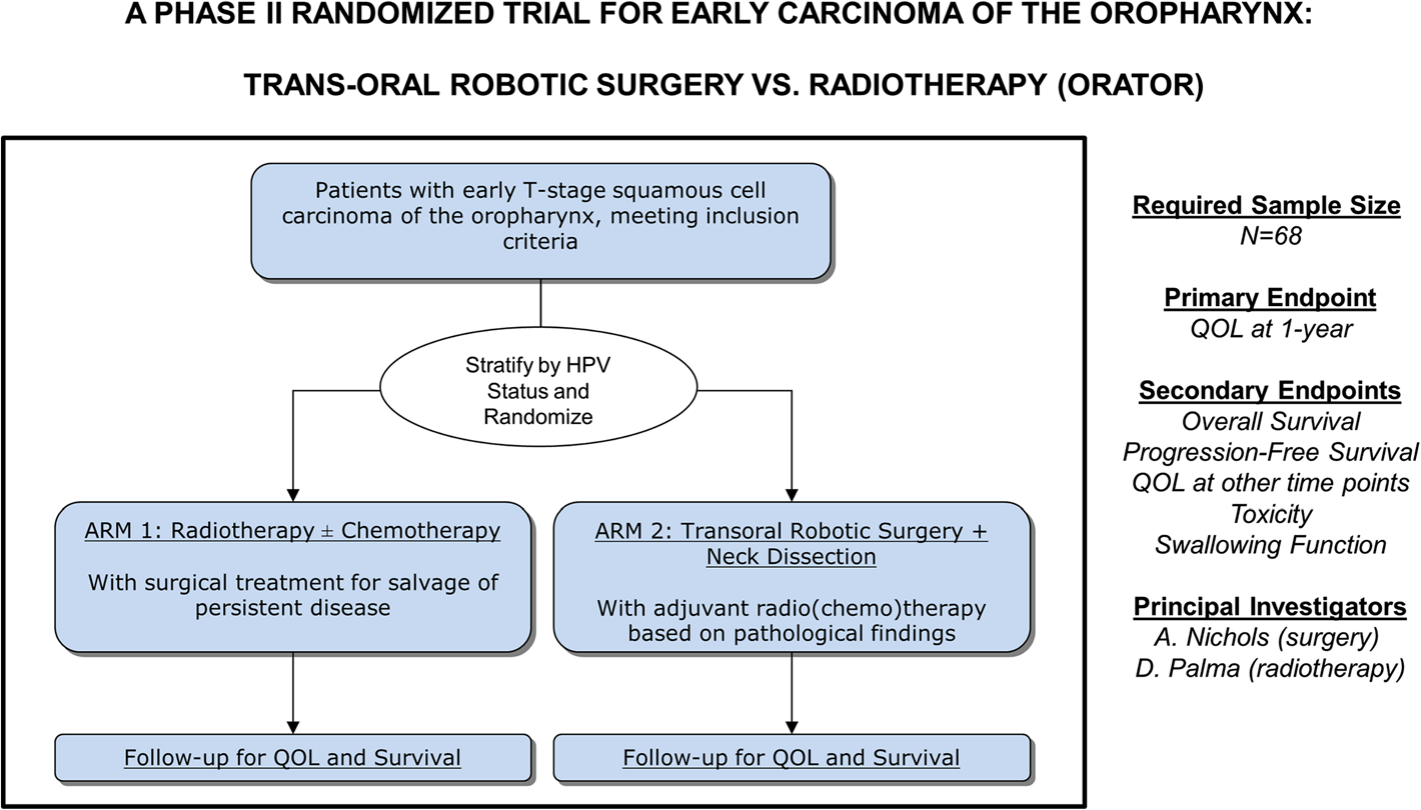
**Study schema. **HPV: human papilloma virus; quality of life: QOL.

**Figure 2:**
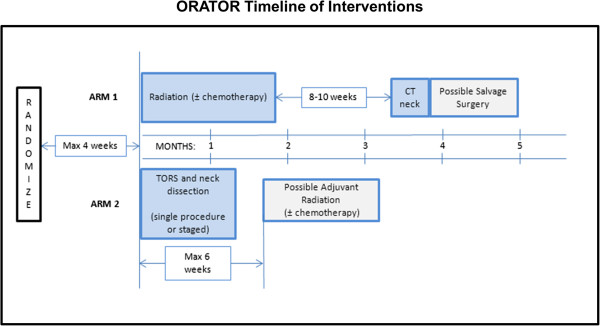
Timeline of interventions.

To compare QOL after primary radiotherapy [± chemotherapy] (Arm 1) vs. TORS [± adjuvant (chemo)radiotherapy] (Arm 2) in patients with squamous cell carcinoma of the oropharynx, to compare toxicity profiles, and to conduct a non-definitive comparison of survival between the two modalities. (See Figure 1 - Study Schema and Figure 2- Timeline of Interventions)

#### Primary endpoint

• QOL 1-year post-treatment

 ○ Assessed with the MD Anderson Dysphagia Inventory (MDADI)

#### Secondary endpoints

• Overall Survival

 ○ Defined as time from randomization to death from any cause

• Progression-free survival

 ○ Time from randomization to disease progression at any site or death

• QOL at other time points

 ○ Using the MD Anderson Dysphagia Inventory (MDADI), the EORTC QLQ-C30 and H&N35 scales, the Voice Handicap Index (VHI-10), the Neck Dissection Impairment Index (NDII), and the Patient Neurotoxicity Questionnaire (PNQ).

• Toxicity

 ○ Assessed by the National Cancer Institute Common Toxicity Criteria (NCI-CTC) version 4

• Swallowing function, measured by:

 ○ Feeding tube rate at 1-year

 ○ MDADI

 ○ CTC-AE Dysphagia scores

#### Inclusion criteria

• Age 18 or older, providing informed consent

• ECOG performance status 0–2

• Histologically confirmed squamous cell carcinoma

• Primary tumor site in the oropharynx (includes tonsil, soft palate, base of tongue, walls of oropharynx)

• Tumor stage: T1 or T2, with likely negative resection margins at surgery

• Nodal stage: N0, N1 (≤ 3 cm), or N2 (between 1–3 cm, on either side of the neck), without extranodal extension on pre-randomization imaging.

• CBC/differential obtained within 4 weeks prior to randomization, with adequate bone marrow function, hepatic, and renal function, defined as: Hemoglobin > 80 g/L; Absolute neutrophil count >1.5 × 10^9^ /L, platelets > 100 × 10^9^/L; Bilirubin < 35 umol/L; AST or ALT < 3 × the upper limit of normal; serum creatinine < 130 umol/L or creatinine clearance ≥ 50 mL/min

• Patient assessed at head and neck multidisciplinary clinic (with assessment by radiation oncologist and surgeon) and presented at multidisciplinary tumor board prior to randomization.

#### Exclusion criteria

• Serious medical comorbidities or other contraindications to radiotherapy, chemotherapy or surgery

• Prior history of head and neck cancer within 5 years

• Prior head and neck radiation at any time

• Metastatic disease

• Inability to attend full course of radiotherapy or follow-up visits

• Prior invasive malignant disease unless disease-free for at least 5 years or more, with the exception of non-melanoma skin cancer

• Unable or unwilling to complete QoL questionnaires

• Pregnant or lactating women

#### Evaluation

The following evaluations are required:

• History and physical examination by a radiation oncologist and head and neck surgeon within 8 weeks prior to randomization, including laryngopharyngoscopy.

• Staging within 12 weeks prior to randomization:

 ○ Contrast-enhanced CT of the head, neck, and chest

or

 ○ MRI of the head and neck with CT of the chest

• Histological confirmation of squamous cell carcinoma

• p16 or HPV tumor status

• CBC/differential, hepatic and renal function testing within 4 weeks of randomization

• Pregnancy test for women of child-bearing age within 2 weeks of randomization

• Dental evaluation within 6 weeks of randomization

• Assessment of dysphagia using CTC-AE version 4 within 2 weeks of randomization

• Completion of QOL scoring within 2 weeks of randomization

• Blood sample for mutational and copy number variation analysis (see section) within 4 weeks of randomization

• Signing of consent within 2 weeks of randomization

• Audiogram before initiation of treatment

### Treatment plan

#### Arm 1: Radiotherapy

Intensity modulated radiotherapy (IMRT) will be used for all patients in this study. IMRT can be delivered using fixed-gantry techniques or rotational techniques (e.g. Tomotherapy or Volumetric Modulated Arc Therapy [VMAT]). Doses and fractionations are shown in Table 1.

**Table 1:** Radiotherapy doses and fractionations

**Target volume**	Radical-intent dose* (Arm 1)	**Adjuvant dose (Arm 2)**
Gross tumor and nodes	70 Gy in 35 fractions over 7 weeks	N/A
Region of positive margins or extra-nodal extension	N/A	64 Gy in 30 fractions over 6 weeks
High-risk nodal areas (and operative bed in Arm 2)	63 Gy in 35 fractions over 7 weeks	60 Gy in 30 fractions over 6 weeks
Low-risk nodal areas	56 Gy in 35 fractions over 7 weeks	54 Gy in 30 fractions over 6 weeks

*In Arm 1, accelerated or hyperfractionated regimens may be used at the discretion of the radiation oncologist (e.g. same doses but delivered in 6 weeks).

### Immobilization and localization

All patients will be immobilized in a custom thermoplastic shell and will undergo a planning CT simulation encompassing the head and neck to below the clavicles. Contrast will be used (unless contra-indicated) for patients in Arm 1. For patients in both arms, the planning CT will be fused with other diagnostic imaging (e.g. MRI scans or pre-operative CT scans for patients in Arm 2) where necessary.

### Radiotherapy volume definitions

#### Arm 1

The gross tumor volume (GTV) is defined as the tumor and any nodes that are either: 1 cm or more in short axis, necrotic, PET positive (where applicable) or biopsy-proven to contain carcinoma. A 5 mm expansion will be added to create the clinical target volume (CTV) CTV70 (excluding natural boundaries of spread), with a further 5 mm to create the planning target volume (PTV) PTV70, which will be treated to a dose of 70 Gy in 35 fractions.

An optional high-risk nodal volume (CTV63) may be defined for areas of uncertainty (e.g. suspicious nodes not meeting the criteria above). A 5 mm margin will be added to create the PTV63, which will be treated to a dose of 63 Gy in 35 fractions.

A lower-risk nodal volume (CTV56) will be defined to include the standard lymphatic drainage sites. For well-lateralized, node-negative tonsillar primaries, this can be limited to the ipsilateral neck; for primaries at the tongue base, this must include both sides of the neck. For patients with a positive node, the contralateral neck must be treated. A 5 mm margin will be added to create the PTV56, which will be treated to a dose of 56 Gy in 35 fractions.

In Arm 1, accelerated or hyperfractionated regimens may be used at the discretion of the radiation oncologist (e.g. same doses but delivered in 6 weeks by delivering 5 twice-daily (BID) treatments, maximum one BID treatment per week)

#### Arm 2

The highest-risk volume (CTV64) will consist of regions of positive margins or extra-nodal extension. A 5 mm margin will be added to create the PTV64, which will be treated to a dose of 64 Gy in 30 fractions.

An intermediate-risk volume (CTV60) will be defined to include the entire tumor bed. CTV60 will also include the pathologically-positive neck. If both sides of the neck are pathologically positive, then both are treated to 60 Gy. A 5 mm margin will be added to create the PTV60, which will be treated to a dose of 60 Gy in 30 fractions.

A lower-risk nodal volume (CTV54) can be defined to include the standard lymphatic drainage sites that have not been dissected and/or low-risk dissected sites. For patients with a positive node, this will include the contralateral neck. A 5 mm margin will be added to create the PTV54, which will be treated to a dose of 54 Gy in 30 fractions.

### Dose constraints

Target dose constraints are shown in Additional file 1: Appendix 1, adapted from RTOG protocols 1016 (Arm 1) and 0920 (Arm 2) [13], and the NCIC-CTG HN6 protocol. Dose constraints are the same for both arms as the radiobiological conversion factor is small.

### Radiotherapy planning

Intensity modulated radiotherapy (IMRT) will be used for all patients in this study. IMRT can be delivered using fixed-gantry techniques or rotational techniques (e.g. Tomotherapy or Volumetric Modulated Arc Therapy [VMAT]). All plans will be normalized to ensure that 95% of each PTV is covered by 95% of the prescription dose for that volume. The maximum dose to PTV70 (Arm 1) and PTV64 (Arm 2) should not exceed 110% of the prescribed dose, and no volume >1 cc outside of these two PTVs should receive >105% of the prescription dose.

### Radiotherapy quality assurance

In order to ensure patient safety and effective treatment delivery, a robust quality assurance protocol is incorporated. The following requirements must be completed for each patient:

• Prior to treatment, each radiotherapy plan will be discussed at head and neck quality assurance (QA) rounds.

• All dose delivery for intensity-modulated plans (including arc-based treatments) will be verified with phantom measurements before treatment by physics staff.

• Cone-beam CT will be used to verify patient positioning immediately prior to treatment at minimum on a weekly basis, with orthogonal x-rays used on other days.

### Concurrent chemotherapy in arm 1

Patients with T1-T2N0 tumors will receive radiotherapy alone, and patients with T1-2 N1-2 tumors will receive radiotherapy with concurrent systemic therapy. Chemotherapy will consist of cisplatin 100 mg/m^2^ delivered every 3 weeks, in 3-week cycles. For patients who are deemed unfit for high dose cisplatin, the dose and/or schedule can be modified, or cetuximab or weekly carboplatin AUC 1.5 can be used, at the discretion of the medical oncologist.

### Salvage surgery in arm 1

Treatment response will be evaluated 8–10 weeks after completion of radiation therapy. For patients with residual nodes > 1 cm in size, a salvage neck dissection will be done. For patients with relapse or progressive disease at any time subsequent to radiation treatment, surgical salvage will be offered if feasible.

### Arm 2: TORS

#### TORS for the primary tumor

For patients with easily accessible oropharyngeal tumors as determined by the consulting surgeon, they will proceed directly to transoral robotic surgery. For patients where adequate transoral access is in question, they will first undergo an examination under anesthesia prior to randomization to ensure adequate exposure can be obtained.

TORS will be carried out using the da Vinci surgical robot (Intuitive Surgical, Sunnyvale, CA, USA). The spatula cautery will be used to remove the tumors with 1 cm margins. At the time of surgery circumferential margins will be taken and sent for frozen section analysis. The resection will proceed until negative margins are obtained if feasible. The learning curve for surgeons carrying out TORS resections has been demonstrated to be short for early-stage cases, likely fewer than 10 cases, with improvements in operative time (but not oncologic outcomes) evident as learning occurs [14]. As a result, surgeons will be required to have carried out at least 10 TORS operations prior to enrolling patients on this trial. If a positive or close margin is found on the final pathology from the TORS resection, an attempt to clear the margin transorally may be performed within four weeks of the original TORS resection. This can be done with or without the robot at the surgeon’s discretion.

### Neck dissection

Patients will undergo standard selective neck dissections for the lymph node areas at risk at the time of TORS, or as a staged procedure within three weeks of the primary site resection, at the discretion of the surgeon. Patients with tonsillar, lateral pharyngeal and lateral palate cancers will undergo ipsilateral neck dissections only, while all other patients will undergo bilateral neck dissections. If levels 1 or 5 are involved they will be dissected, otherwise selective neck dissections will be limited to levels 2–4. For patients with positive margins at the primary site at the time of TORS, an attempt can be made at the time of neck dissection to clear the positive margin transorally.

### Adjuvant radiotherapy and chemotherapy

Adjuvant radiotherapy and chemotherapy will be delivered in accordance with National Comprehensive Cancer Network (NCCN) Clinical Guidelines [15]. Radiotherapy prescriptions and planning details are outlined above.

*Radiotherapy can be omitted* if there are no adverse pathological features (i.e. none of the following: extranodal extension, positive margins, pT3 or pT4 disease, nodal disease, or lymphovascular invasion).

*Adjuvant radiotherapy alone* is recommended for the following risk factors:

• Any node positive

• Lymphovascular invasion

• pT3 or pT4 tumor

• Close resection margins (<2 mm)

For patients with perineural invasion (PNI) alone and no other indications for radiotherapy, radiotherapy will be omitted [16].

*Adjuvant chemotherapy concurrent with radiotherapy* is required for patients with positive margins or extra-capsular extension. The chemotherapy will consist of cisplatin 100 mg/m^2^ delivered every 3 weeks, in 3-week cycles. For patients who are deemed unfit for such chemotherapy, the dose and/or schedule can be modified, or weekly carboplatin AUC 1.5 can be utilized, at the discretion of the medical oncologist.

### Follow-up evaluation and assessment of efficacy

The follow-up schedule is shown in Additional file 2: Appendix 2. Day 1 of follow-up will be the first day of radiotherapy (Arm 1) or the date of surgery (Arm 2); however, survival will be calculated from the date of randomization.

Arm 1: For patients receiving radiotherapy, they will be seen weekly during radiotherapy, and 4–6 weeks after radiotherapy, as part of routine care. A CT scan of the neck will be carried out 8–10 weeks after radiotherapy for assessment of residual disease for neck dissection, with a routine radiotherapy appointment 2 weeks after the CT scan (approximately 4 months from the start of treatment).

Arm 2: Patients will be seen approximately 2 weeks after completion of the neck dissection for routine post-operative assessment. Adjuvant radiotherapy, if required, shall begin within 6 weeks of surgery. Radiotherapy will be pre-booked to start within 6 weeks of the date of TORS to avoid unnecessary delays. During radiotherapy, routine visits will occur weekly during radiotherapy and 4–6 weeks afterward. For all patients in Arm 2, a return visit with the surgeon will occur at 3 months from the date of TORS.

Both arms: In addition to the above, patients will be seen at 3, 6, 9, 12, 15, 18, 21, 24, 30, 36, 42, 48, 54, and 60 months from first date of treatment. At each visit, a history and physical examination will be conducted (including laryngopharyngoscopy), and CTC-AE toxicities recorded. The QOL questionnaires are to be completed every 6 months except for the Patient Neurotoxicity Questionnaire (PNQ) that will be completed one year post-treatment. A chest x-ray will be completed every 6 months (except at the 12 month visit when a CT chest is undertaken). One year post-randomization, an audiogram should be performed.

For patients in both arms, a CT of the head, neck, and chest will be done at 12 months. Additional imaging or laboratory investigations should be carried out at the discretion of the oncologist, based on findings in the history or physical, and additional treatment (e.g. salvage treatment) is at the discretion of the treating physicians. During treatment, blood tests will be performed as per standard of care and highest BUN and creatinine values and lowest white blood cell, neutrophil and platelet counts will be recorded. One year post-treatment, a blood test to measure BUN, creatinine, and CBC/differential will be performed.

### Measurement of response

Survival outcomes: Overall survival will be measured as time from randomization until death from any cause, and progression-free survival as time to either progression or death, whichever occurs first.

QOL outcomes: The MDADI, EORTC scales, NDII, and VHI-10 will be measured at baseline and at 6-month intervals. PNQ will be completed at 1 year post-treatment.

Toxicity outcomes: CTC-AE toxicities will be recorded at every follow-up visit (3, 6, 9, 12, 15, 18, 21, 24, 30, 36, 42, 48, 54, and 60 months from the first date of treatment).

### Quality assurance for sites joining trial

The “learning curve” associated with TORS has been reported as a minimum of approximately 10 cases, with improvements in operating time noted after such learning is complete [14]. Importantly, such learning did not appear to affect the rates of positive margins. Prior to opening the study, in addition to local research ethics approval, each surgeon enrolling patients onto trial must have completed at least 10 previous TORS cases, and one such case will be proctored by one of the Principal Investigators (ACN). Each centre will be required to complete mock radiotherapy treatment plans, to ensure that such plans are designed in compliance with the protocol. The principal investigators will provide pertinent CT datasets. There will be three such mock plans: one node-negative radical case, one node-positive radical case, and one adjuvant case. Once these have been received and approved, the centre can be activated. Once the study is open, each centre may enroll and treat patients. For each patient enrolled on study and receiving radiation therapy, an electronic copy of the full radiation treatment plan must be sent to the principal investigators on or before the first day of treatment. Radiotherapy treatment can begin, but the plan will be reviewed within 5 business days to ensure there are no major deviations from the protocol.

### Statistics and sample size calculation

The study will employ a 1:1 randomization between Arm 1:Arm 2 (Figure 1) in a permutated block design. This sample size allows for one stratification factor at randomization, which will be HPV status: (positive vs. negative/indeterminate)

### Sample size considerations

This study will employ a randomized phase II screening design, to conduct a definitive QOL comparison as the primary endpoint, along with preliminary and non-definitive overall survival comparison between the two arms as a secondary endpoint [17]. The primary endpoint is the total score on all twenty items of the MDADI. It is generally believed that a 10-point difference in standardized QOL scores represents a clinically significant difference in QOL [18]. It is assumed that the QOL scores will be normally distributed with a standard deviation of 12. The study will use a two-sided, independent-sample t-test with an alpha level of 0.05 and power of 90%, and assume a dropout rate of 10%. There will be a 1:1 randomization between Arm 1 and Arm 2. In order to detect a 10-point improvement in QOL in the TORS arm (Arm 2), a total of 68 patients will be required (34 in each arm). Accrual targets are as follows: 10 patients in year 1, and 15–20 patients in years 2, 3, and 4.

### Analysis plan

Patients will be analyzed in the groups to which they are assigned (intention-to-treat).

An independent-sample t-test will be used to compare QOL scores at 1-year on the MDADI. The percent of patients in each arm who experience a clinically significant QOL decline (10 points) will also be reported. Survival will be calculated from date of randomization using the Kaplan-Meier method with differences compared using the log-rank test. Pre-planned subgroup analysis will occur based on the stratification variable. A Cox multivariable regression analysis will be used to determine baseline factors predictive of survival. For the secondary endpoints involving QOL scales, linear mixed effects models will be used; for the MDADI, NDII and VHI-10, the total scores will be compared between the two arms, whereas for the EORTC scales, each of the subscales (e.g. pain, swallowing, etc.) will be compared between the two arms. The PNQ scores (A to E) will be converted to a numerical score (0 to 4, respectively), and the mean scores in each group will be compared with a t-test. In addition, the proportion of patients reporting severe neurological dysfunction (D or E) will be compared with the Chi-Squared Test, or Fisher's exact test, as appropriate.

### Data Safety Monitoring Committee (DSMC)

The DSMC, consisting of at least one radiation oncologist, one medical oncologist, and surgical oncologist not involved in the study, will meet quarterly after study initiation to review toxicity outcomes. If any grade 3–5 toxicity is reported, the DSMC will review the case notes to determine if such toxicity is related to treatment. If the DSMC deems that toxicity rates are excessive (e.g. >5% grade 4 or 5 toxicity), then the DSMC can, at its discretion, recommend modification or cessation of the trial.

*Interim analysis*: the DSMC will conduct one interim analysis once 30 patients have been accrued and followed for 1-year. For this analysis, the DSMC will be blinded to the identity of each treatment arm, but QOL and OS data will be presented for each arm.

The DSMC will recommend stopping the trial if there is an OS difference that is statistically significant with a threshold of p < 0.001 using the log-rank test, based on the Haybittle-Peto stopping rule; this retains an overall alpha of 0.05. Furthermore, if the QOL scores among all patients are substantially different than estimated in the sample size calculation, the DSMC can recommend increasing or decreasing the target accrual in order to maintain statistical power.

### Biomarker studies

#### Human papillomavirus testing

P16 testing (which is an excellent surrogate marker of HPV status) is required for stratification at the time of randomization. This will be done through the routine pathology laboratories as per current routine clinical care.

The accompanying biomarker study will determine HPV status by real-time polymerase chain reaction (PCR), not for the purposes of randomization, but to confirm the accuracy of P16 results and also for subtyping of HPV strain. For patients randomized to radiotherapy, pre-treatment formalin fixed paraffin embedded (FFPE) primary site biopsy specimens will be retrieved in 10 slides 8um thick from the FFPE blocks. For patients randomized to TORS, the main specimen will be taken to pathology frozen section room and a portion from the center of the specimen will be taken with the assistance of the pathologist and frozen at -80 degrees Celsius. DNA will be extracted from the specimens for HPV testing by real-time PCR.

### Mutational and copy number variation analysis

DNA will be extracted either from formalin fixed specimens or preferably fresh tumor for patients undergoing TORS as well as 10 mL of venous blood drawn prior to the initiation of treatment. Specimens yielding DNA of adequate quantity and quality (>5 μg, OD between 1.8 and 2.0) will be subjected to high-throughput sequencing and gene copy number analysis.

## Discussion

In the wake of the HPV oropharyngeal cancer epidemic, it is imperative that we have treatment strategies that optimize post-treatment QOL for these younger, healthier patients. Radiation therapy (+/- chemotherapy) has been proven highly effective and provides a high QOL for most patients; however, a subset can suffer significant toxicity including severe dysphagia requiring gastrostomy tube dependence. Although the preliminary outcomes of TORS are encouraging, rapid adoption has occurred in the United States without clear evidence that it provides equivalent or superior QOL and survival outcomes. It is critical that this question be answered in a systematic way to either support this change in treatment strategy or halt this transition. The results of this study have the potential to guide improvements in the QOL of patients with OPSCC in Canada and worldwide.

The implementation of new technologies in medicine often follows Roger’s innovation adoption life-cycle: after the technique is developed by innovators, it is taken up by early adopters, followed by the early- and late- majority, and then laggards in a bell-curve distribution [19]. The ideal time to test a new innovation is at the early-adoption/early majority phase, as after a technique is widely adopted, benefits can be assumed (sometimes erroneously) and equipoise is lost [20]. TORS is at this juncture in Canada, with early adopters having implemented the technique, with several other centres planning to implement the technology over the next few years. This provides a key opportunity to test the benefits of TORS.

Identification of biomarkers of radiation or surgical failure would be highly useful to guide patients toward therapies with the highest chance of cure while avoiding unnecessary toxicity. A randomized trial is the ideal setting for biomarker discovery as confounding factors are minimized and follow-up is robust. In our study, the primary tumor and blood samples will undergo whole exome sequencing with copy number analysis. Although the study is not powered for biomarker discovery, the next generation analysis may reveal interesting genetic findings comparing HPV positive and negative cancers. Should a phase III trial be warranted, biomarker discovery will be an integral component in order to identify individuals who would benefit the most from either modality or are at high-risk of treatment related toxicity such as ototoxicity. Reliable biomarkers would represent a tremendous step towards customizing care for the increasing number of patients suffering with OPSCC.

## Competing interests

The authors declares that they have no competing interests.

## Authors’ contributions

Study conception and design: all authors. Draft of original protocol: ACN and DAP. Critical revision of protocol: All authors have read and approve the final manuscript.

## Pre-publication history

The pre-publication history for this paper can be accessed here:

http://www.biomedcentral.com/1471-2407/13/133/prepub

## Supplementary Material

Additional file 1: Appendix 1 Normal Tissue Dose Constraints. Click here for file

Additional file 2: Appendix 2 Follow-up Schedule. Click here for file

## References

[B1] Chaturvedi AK Engels EA Pfeiffer RM Hernandez BY Xiao W Kim E Jiang B Goodman MT Sibug-Saber M Cozen W Human papillomavirus and rising oropharyngeal cancer incidence in the United States J Clin Oncol 2011 29 32 4294 4301 10.1200/JCO.2011.36.4596 21969503 PMC3221528

[B2] Parsons JT Mendenhall WM Stringer SP Amdur RJ Hinerman RW Villaret DB Moore-Higgs GJ Greene BD Speer TW Cassisi NJ Squamous cell carcinoma of the oropharynx: surgery, radiation therapy, or both Cancer 2002 94 11 2967 2980 10.1002/cncr.10567 12115386

[B3] Haigentz M Jr Silver CE Corry J Genden EM Takes RP Rinaldo A Ferlito A Current trends in initial management of oropharyngeal cancer: the declining use of open surgery Eur Arch Otorhinolaryngol 2009 266 12 1845 1855 10.1007/s00405-009-1109-2 19866522

[B4] Pignon JP Le Maitre A Maillard E Bourhis J Meta-analysis of chemotherapy in head and neck cancer (MACH-NC): an update on 93 randomised trials and 17,346 patients Radiother Oncol 2009 92 1 4 14 10.1016/j.radonc.2009.04.014 19446902

[B5] Machtay M Moughan J Trotti A Garden AS Weber RS Cooper JS Forastiere A Ang KK Factors associated with severe late toxicity after concurrent chemoradiation for locally advanced head and neck cancer: an RTOG analysis J Clin Oncol 2008 26 21 3582 3589 10.1200/JCO.2007.14.8841 18559875 PMC4911537

[B6] Dowthwaite SA Franklin JH Palma DA Fung K Yoo J Nichols AC The role of transoral robotic surgery in the management of oropharyngeal cancer: a review of the literature Isrn Oncology 2012 2012 945162 22606380 10.5402/2012/945162 PMC3347745

[B7] Leonhardt FD Quon H Abrahao M O'malley BW Jr Weinstein GS Transoral robotic surgery for oropharyngeal carcinoma and its impact on patient-reported quality of life and function Head Neck 2012 34 2 146 154 10.1002/hed.21688 21469248

[B8] Calais G Alfonsi M Bardet E Sire C Germain T Bergerot P Rhein B Tortochaux J Oudinot P Bertrand P Randomized trial of radiation therapy versus concomitant chemotherapy and radiation therapy for advanced-stage oropharynx carcinoma J Natl Cancer Inst 1999 91 24 2081 2086 10.1093/jnci/91.24.2081 10601378

[B9] Setton J Caria N Romanyshyn J Koutcher L Wolden SL Zelefsky MJ Rowan N Sherman EJ Fury MG Pfister DG Intensity-modulated radiotherapy in the treatment of oropharyngeal cancer: an update of the memorial Sloan-Kettering cancer center experience Int J Radiat Oncol Biol Phys 2012 82 1 291 298 10.1016/j.ijrobp.2010.10.041 21167652

[B10] Sinclair CF Mccolloch NL Carroll WR Rosenthal EL Desmond RA Magnuson JS Patient-perceived and objective functional outcomes following transoral robotic surgery for early oropharyngeal carcinoma Arch Otolaryngol Head Neck Surg 2011 137 11 1112 1116 10.1001/archoto.2011.172 22106235

[B11] Bosco LF Silliman RA Thwin SS Geiger AM Buist DSM Prout MN Yood MU Haque R Wei F Lash TL A most stubborn bias: No adjustment method fully resolves confounding by indication in observational studies J Clin Epidemiol In Press, Corrected Proof 10.1016/j.jclinepi.2009.03.001 PMC2789188 19457638

[B12] Cohen MA Weinstein GS O'malley BW Jr Feldman M Quon H Transoral robotic surgery and human papillomavirus status: oncologic results Head Neck 2011 33 4 573 580 10.1002/hed.21500 21425382

[B13] Radiation Therapy Oncology Group Active protocols[http://www.rtog.org]

[B14] Lawson G Matar N Remacle M Jamart J Bachy V Transoral robotic surgery for the management of head and neck tumors: learning curve Eur Arch Otorhinolaryngol 268 12 1795 1801 21365213 10.1007/s00405-011-1537-7

[B15] Nccn Clinical Practice Guidelines [http://www.nccn.org]

[B16] Liao CT Chang JT Wang HM Ng SH Hsueh C Lee LY Lin CH Chen IH Huang SF Cheng AJ Does adjuvant radiation therapy improve outcomes in pT1-3N0 oral cavity cancer with tumor-free margins and perineural invasion? Int J Radiat Oncol Biol Phys 2008 71 2 371 376 10.1016/j.ijrobp.2007.10.015 18474310

[B17] Rubinstein LV Korn EL Freidlin B Hunsberger S Ivy SP Smith MA Design issues of randomized phase II trials and a proposal for phase II screening trials J Clin Oncol 2005 23 28 7199 7206 10.1200/JCO.2005.01.149 16192604

[B18] Ringash J O'sullivan B Bezjak A Redelmeier DA Interpreting clinically significant changes in patient-reported outcomes Cancer 2007 110 1 196 202 10.1002/cncr.22799 17546575

[B19] Rogers E Diffusion of innovations 2003 5 New York, USA: Free Press

[B20] Mcculloch P Altman DG Campbell WB Flum DR Glasziou P Marshall JC Nicholl J Aronson JK Barkun JS Blazeby JM No surgical innovation without evaluation: the IDEAL recommendations Lancet 2009 374 9695 1105 1112 10.1016/S0140-6736(09)61116-8 19782876

