# Supplementary material for: Early-stage squamous cell carcinoma of the oropharynx: Radiotherapy vs. Trans-Oral Robotic Surgery (ORATOR) – study protocol for a randomized phase II trial
Source: BMC Cancer. 2013 Mar 20;13:133. doi: 10.1186/1471-2407-13-133 (PMC3621077; doi:10.1186/1471-2407-13-133)
Supplement: Additional file 1: Appendix 1 — Normal Tissue Dose Constraints. [file 1471-2407-13-133-S1.doc]

**Appendix 1: Normal Tissue Dose Constraints**

| **Structure** | **Maximum dose for either Arm 1 or Arm 2** |
| --- | --- |
| Spinal Cord | 48 Gy point dose  45 Gy to 0.1 cc |
| Spinal Cord PRV  (defined as spinal cord + 5 mm) | 52 Gy to 0.1 cc |
| Brainstem | 54 Gy point dose  50 Gy to 0.1 cc |
| Brainstem PRV  (defined as brainstem + 5 mm) | 60 Gy to 0.1 cc |
| Lips | Mean < 20 Gy |
| Oral Cavity* | Mean < 30 Gy |
| Parotid* | Mean < 26 Gy |
| Mandible* | Maximum < 66 Gy |
| Larynx* | Maximum <45 Gy |

*Maximum doses will be exceeded if the PTV overlaps with,

or is in close proximity to, these structures
