# Supplementary material for: Early-stage squamous cell carcinoma of the oropharynx: Radiotherapy vs. Trans-Oral Robotic Surgery (ORATOR) – study protocol for a randomized phase II trial
Source: BMC Cancer. 2013 Mar 20;13:133. doi: 10.1186/1471-2407-13-133 (PMC3621077; doi:10.1186/1471-2407-13-133)
Supplement: Additional file 2: Appendix 2 — Follow-up Schedule. [file 1471-2407-13-133-S2.doc]

**Appendix 2 – Followup Schedule**

**Follow-up dates calculated from first day of treatment**

| **YEAR 1** | **Before**  **Entry** | **Month**  **3*** | **Month**  **4** | **Month**  **6** | **Month**  **9** | **Month**  **12** |
| --- | --- | --- | --- | --- | --- | --- |
| History and Physical | X | X |  | X | X | X |
| Baseline staging investigations  (see section 5.0) | X |  |  |  |  |  |
| QOL scoring | X |  |  | X |  | X |
| Toxicity Scoring | X | X |  | X | X | X |
| Follow-up CT head and neck to assess for residual nodes post-RT  (Arm 1) |  |  | X |  |  |  |
| Follow-up CT head, neck and chest  (Both Arms) |  |  |  |  |  | X |
| Follow-up chest x-ray |  |  |  | X |  |  |
| Audiogram | X |  |  |  |  | X |
| BUN, Creatinine, and CBC/Differential during treatment and at 12 months | X |  |  |  |  | X |

*Some patients will still be on treatment at Month 3

| **YEARS 2-5** | **Month 15** | **Month**  **18** | **Month**  **21** | **Month 24 and every 6 months thereafter until 5 years** |
| --- | --- | --- | --- | --- |
| History and Physical | X | X | X | X |
| QOL scoring |  | X |  | X |
| Toxicity Scoring | X | X | X | X |
| Follow-up chest x-ray |  | X |  | X |
